# First estimation of direct H1N1pdm virulence: From reported non consolidated data from Mauritius and New Caledonia

**DOI:** 10.1371/currents.RRN1010

**Published:** 2009-08-23

**Authors:** Antoine Flahault

**Affiliations:** EHESP School of Public Health

## Abstract

We provide rough estimates of direct lethality from ARDS due to H1N1pdm from two independent sources of data, one from New Caledonia where 30,000 infections are assumed to have occured and 3 deaths reported to be attributable directly to the pandemic virus. Another source is Mauritius where 70,000 infections are estimated to have occured, and 7 reported death from ARDS (5 of them are currently confirmed). These surveillance data allows for first estimation of direct lethality due to H1N1pdm to be 1 per 10,000 infections, about 100 times more than regular seasonal influenza.

Influenza lethality is due to three main causes: First, direct viral origin, leading to viral pneumonia of high severity, with an acute respiratory distress syndrom (ARDS), associated with 30 to 50% lethality in intensive Care Unit (ICU) [1]; Second, bacterial surinfection, due to pneumococal, staphylococal, streptococal, or meningococal pneumonia, usely curable with appropriate antibiotics, provided they are administered early enough; and Third, decompensation of severe underlying conditions, often in elderly people or in vulnerable chronic patients. The last one is the predominant cause of death attributable to seasonal influenza in developed countries, and has reached for years now, despite of the use of mass immunization in elderly, a level around 1 death per 1,000 infections. This cause of lethality is rarely reported as attributed to influenza in death certificates, and therefore is not easily assessable during outbreaks, and is usely measured as excess mortality when time series of mortality become available, sometimes in real time, most often several months after the season [2]. The second cause, i.e. bacterial surinfections, is much less seen in developed countries due to antibiotics availability. However, in the developing world, it may still be a cause of concern, since bacterial pneumonia may rapidely become lethal if treatment is not given appropriately in time. The direct lethality due to viral pneumonia gives probably the best estimate of influenza strains' virulence, since it may vary from strain to strain, and is not due to the level of health development of a country. Furthermore, ARDS always leads to ICU, and is easy enough to diagnose and to report.

Death from ARDS due to seasonal influenza

There are not many assessments available with regards with the incidence of death from ARDS due to seasonal influenza in the litterature. Empirical evidence suggests in France that less than 5 to 10 of such cases are identified each year, when an average of 6 million seasonal infections are estimated from the French Sentinelles system [3]. We may therefore assume, waiting for better estimates in the future, that deaths from ARDS due to seasonal influenza is an exceptional event, which occurs once every million infected patients.

Death from ARDS due to H1N1pdm

It is too early to provide precise figures of deaths of ARDS due to the new strain of pandemic influenza. In the USA there are a too large uncertainty reported on the denominator (i.e. number of influenza infections), when in the UK or Argentina too. In smaller areas, and in particular in islands such as French New Caledonia or Mauritius, we may have first rough estimates which may be more relevant and easier to catch.

French Government estimated last week that 20,000 cases of influenza had occured in New Caledonia in the emerging outbreak, leading to a rough estimate of 30,000 infections and by August 21st, 3 deaths of confirmed cases were reported, in patients aged 8 yr, 27 yr, 58 yr [4].

Mauritius Government estimated last week that 15,000 cases of influenza had occured in the last two weeks in  Mauritius, however some reports from the media and recent personal communication from local doctors lead me to estimate that about 50,000 cases actually occured, and therefore about 70,000 infections, taking acount mild illnesses. By August 19th, 7 deaths had occured, 5 of them were already virologically confirmed in patients aged 4, 6, 28, 46, 53 [5].

New Caledonia and Mauritius figures are consistant to a rough estimate of about 1 death from ARDS due to H1N1pdm per 10,000 infections, i.e. a virulence of an order of magnitude of 100 times that observed for seasonal strains.

We acknowledge that we are producing very rough estimates of direct lethality associated to H1N1pdm. However, it may be useful to deliver such estimates as early as possible in the course of this pandemic, with the purpose of helping health authorities to check availability of ICUs and artificial ventilation devices in their countries, in case of a wave of similar virulence this fall in the Northern Hemisphere. When assuming that half of ARDS are of lethal outcome, we may assume that H1N1pdm may generate 1 case of ARDS every 5,000 infections. ARDS is always treated in ICUs, and three weeks of treatment is probably an average period to be taken into account. In case of attack rates of 30% to 50% this fall and winter in Northern Hemisphere [6], these figures may help refining preparedness.

This work is funded by EHESP School of Public Health, Rennes, FranceThe author has declared that no competing interests exist
